# Nijmegen breakage syndrome (NBS)

**DOI:** 10.1186/1750-1172-7-13

**Published:** 2012-02-28

**Authors:** Krystyna H Chrzanowska, Hanna Gregorek, Bożenna Dembowska-Bagińska, Maria A Kalina, Martin Digweed

**Affiliations:** 1 Department of Medical Genetics, The Children's Memorial Health Institute, Al. Dzieci Polskich 20, 04-730 Warsaw, Poland; 2 Department of Microbiology and Clinical Immunology, The Children's Memorial Health Institute, Al. Dzieci Polskich 20, 04-730 Warsaw, Poland; 3 Department of Oncology, The Children's Memorial Health Institute, Al. Dzieci Polskich 20, 04-730 Warsaw, Poland; 4 Department of Paediatrics, Endocrinology and Diabetes, Medical University of Silesia, ul. Medyków 16, 40-752 Katowice, Poland; 5 Institut für Medizinische Genetik und Humangenetik, Charité-- Universitätsmedizin Berlin, Campus-Virchow Klinikum, Berlin, Germany

**Keywords:** Nijmegen breakage syndrome, Chromosomal instability, Immunodeficiency, Microcephaly, Predisposition to malignancy, Hypergonadotropic hypogonadism

## Abstract

Nijmegen breakage syndrome (NBS) is a rare autosomal recessive syndrome of chromosomal instability mainly characterized by microcephaly at birth, combined immunodeficiency and predisposition to malignancies. Due to a founder mutation in the underlying *NBN *gene (c.657_661del5) the disease is encountered most frequently among Slavic populations. The principal clinical manifestations of the syndrome are: microcephaly, present at birth and progressive with age, dysmorphic facial features, mild growth retardation, mild-to-moderate intellectual disability, and, in females, hypergonadotropic hypogonadism. Combined cellular and humoral immunodeficiency with recurrent sinopulmonary infections, a strong predisposition to develop malignancies (predominantly of lymphoid origin) and radiosensitivity are other integral manifestations of the syndrome. The *NBN *gene codes for nibrin which, as part of a DNA repair complex, plays a critical nuclear role wherever double-stranded DNA ends occur, either physiologically or as a result of mutagenic exposure. Laboratory findings include: (1) spontaneous chromosomal breakage in peripheral T lymphocytes with rearrangements preferentially involving chromosomes 7 and 14, (2) sensitivity to ionizing radiation or radiomimetics as demonstrated *in vitro *by cytogenetic methods or by colony survival assay, (3) radioresistant DNA synthesis, (4) biallelic hypomorphic mutations in the *NBN *gene, and (5) absence of full-length nibrin protein. Microcephaly and immunodeficiency are common to DNA ligase IV deficiency (LIG4 syndrome) and severe combined immunodeficiency with microcephaly, growth retardation, and sensitivity to ionizing radiation due to NHEJ1 deficiency (NHEJ1 syndrome). In fact, NBS was most commonly confused with Fanconi anaemia and LIG4 syndrome. Genetic counselling should inform parents of an affected child of the 25% risk for further children to be affected. Prenatal molecular genetic diagnosis is possible if disease-causing mutations in both alleles of the *NBN *gene are known. No specific therapy is available for NBS, however, hematopoietic stem cell transplantation may be one option for some patients. Prognosis is generally poor due to the extremely high rate of malignancies.

Zespół Nijmegen (*Nijmegen breakage syndrome*; NBS) jest rzadkim schorzeniem z wrodzoną niestabilnością chromosomową dziedziczącym się w sposób autosomalny recesywny, charakteryzującym się przede wszystkim wrodzonym małogłowiem, złożonymi niedoborami odporności i predyspozycją do rozwoju nowotworów.

Choroba występuje najczęściej w populacjach słowiańskich, w których uwarunkowana jest mutacją założycielską w genie *NBN *(c.657_661del5). Do najważniejszych objawów zespołu zalicza się: małogłowie obecne od urodzenia i postępujące z wiekiem, charakterystyczne cechy dysmorfii twarzy, opóźnienie wzrastania, niepełnosprawność intelektualną w stopniu lekkim do umiarkowanego oraz hipogonadyzm hipogonadotropowy u dziewcząt. Na obraz choroby składają się także: niedobór odporności komórkowej i humoralnej, który jest przyczyną nawracających infekcji, znaczna predyspozycja do rozwoju nowotworów złośliwych (zwłaszcza układu chłonnego), a także zwiększona wrażliwość na promieniowanie jonizujące. Wyniki badań laboratoryjnych wykazują: (1) spontaniczną łamliwość chromosomów w limfocytach T krwi obwodowej, z preferencją do rearanżacji chromosomów 7 i 14, (2) nadwrażliwość na promieniowanie jonizujące lub radiomimetyki, co można wykazać metodami *in vitro*, (3) radiooporność syntezy DNA, (4) hipomorficzne mutacje na obu allelach genu *NBN*, oraz (5) brak w komórkach pełnej cząsteczki białka, nibryny. Małogłowie i niedobór odporności występują także w zespole niedoboru ligazy IV (LIG4) oraz w zespole niedoboru NHEJ1. Rodzice powinni otrzymać poradę genetyczną ze względu na wysokie ryzyko (25%) powtórzenia się choroby u kolejnego potomstwa. Możliwe jest zaproponowanie molekularnej diagnostyki prenatalnej jeżeli znane są obie mutacje będące przyczyną choroby. Nie ma możliwości zaproponowania specyficznej terapii, ale przeszczep szpiku może być alternatywą dla niektórych pacjentów. Generalnie prognoza nie jest pomyślna z uwagi na wysokie ryzyko rozwoju nowotworu.

## Disease name and synonyms

Nijmegen breakage syndrome (NBS) (MIM #251260)

Ataxia-telangiectasia variant V1; AT-V1

Microcephaly with normal intelligence, immunodeficiency, and lymphoreticular malignancies (Seemanova syndrome II)

Immunodeficiency, microcephaly, and chromosomal instability

**Berlin breakage syndrome (BBS) **(MIM #602667) synonymous with #251260 Ataxia-telangiectasia variant V2; AT-V2

A synonym given in MIM using the term "nonsyndromal microcephaly" should not be used, as it is misleading.

## Definition

Nijmegen breakage syndrome is a rare autosomal recessive disease presenting at birth with microcephaly but generally no additional neurological manifestations. Other important clinical features, more noticeable with age, include mild growth delay, premature ovarian insufficiency, predisposition to recurrent infections of various organs and a very high risk to develop malignancies at an early age, most frequently of haematological origin. Psychomotor development is usually not disturbed despite progressive microcephaly, however, deterioration of cognitive functions may occur with age. Combined immunodeficiency of both the cellular and humoral response is an essential feature of the disease. Chromosomal instability with characteristic rearrangements in peripheral T lymphocytes in the form of inversions and translocations involving chromosomes 7 and 14, and cellular sensitivity to ionising radiation (IR) *in vitro *are all characteristic for the disease and have diagnostic relevance. Identifying mutations in both alleles of the *NBN *gene (formerly *NBS1*) completes the diagnosis of NBS.

## Historical notes

The first description was in 1979 of a Dutch boy with microcephaly, growth and developmental retardation, IgA deficiency and chromosomal rearrangements resembling those observed in ataxia telangiectasia (A-T), i.e. affecting chromosomes 7 and 14 with breakpoints in four sites (7p13, 7q35, 14q11 and 14q32) [1]. The discovery that a deceased brother of this patient had presented with similar clinical features led in 1981 to the formal description of this genetic disease by researchers at the University of Nijmegen in the Netherlands, who named it, Nijmegen breakage syndrome [2]. Patients manifesting microcephaly with normal intelligence, immunodeficiency, and an unprecedentedly strong predisposition to lymphoreticular malignancies were reported in 1985 as the Seemanova Syndrome II [3] but it was subsequently confirmed that they were actually affected with the same disease [4].

In addition to chromosomal instability, intensive studies of NBS cells *in vitro *showed other cellular features similar to those found in ataxia-telangiectasia, such as sensitivity to IR and radioresistant DNA synthesis (RDS) [4-6]. For these reasons, NBS was considered to be a variant of A-T, even though the neurological symptoms are clearly different and neither ataxia nor telangiectasia are observed in NBS [7]. Complementation studies of different NBS cell lines suggested genetic heterogeneity and two groups were distinguished: A-T variant 1 (AT-V1) or Nijmegen breakage syndrome and A-T variant 2 (AT-V2) or Berlin breakage syndrome [5,8].

In 1998 the gene responsible for NBS, originally designated as *NBS1 *but now renamed as *NBN*, was cloned and the gene product, nibrin, was identified [9-11]. Nibrin, together with MRE11 and RAD50 forms a trimeric protein complex (MRN) involved in repairing DNA double strand breaks (DSBs). It became immediately apparent that mutations in the same gene were responsible for both complementation groups, AT-V1 and AT-V2 [11].

## Epidemiology

Nijmegen breakage syndrome is a rare disease and there are no reliable estimates of its prevalence. The number of known patients identified worldwide increased significantly when the disease-causing gene, *NBN*, was identified. Apart from over 150 subjects reported in the medical literature [12-36], there are many more patients recorded in national registries (e.g. Czech and Polish--CKH p.c.). Currently, the largest European registry is managed by the European Society for Immunodeficiencies (ESID).

The disease seems to occur worldwide, but with a distinctly higher prevalence among Central European and Eastern European populations, i.e. in the Czech Republic, Poland, Russia and Ukraine [3,6,31,34]. The proportion of patients identified in these populations correlates with a high carrier frequency of the major *NBN *mutation, c.657_661del5 (p.K219fsX19), estimated to be 1 case per 177 newborns, clearly the result of a founder effect [37]. Surprisingly, a similarly high frequency of this mutation (1/176) has been found among newborns in Northeast Bavaria, suggesting a relatively high proportion of people of Slavic origin in that region [38].

Nijmegen breakage syndrome has also been reported in many other European countries [2,8,14,23,36,39-42], as well as in North and South America, Morocco and New Zealand [12,15,22,27,32,35,43-46].

## Clinical description

The clinical phenotype of NBS consists of several cardinal features, such as progressive microcephaly, which influences facial phenotype, mild growth delay, premature ovarian failure, cellular and humoral immunodeficiency predisposing to recurrent infections, and an exceptionally high risk of cancer development at an early age.

Clinical manifestations described in detail below are not exclusively specific for NBS, and can also vary in severity.

## Microcephaly and craniofacial features

A hallmark symptom of NBS is microcephaly, which is observed from birth onwards and should alert a neonatologist or a paediatrician [12,47,48]. Microcephaly is defined as a reduction in occipitofrontal circumference (OFC) to below -2 standard deviations (SD) from the mean as compared to a healthy population of the same age and sex. Microcephaly in NBS is progressive and can be as severe as -9 SD in older children [49]. At birth, the anterior fontanel is usually hardly palpable and is closed within the first few weeks of life. Importantly, microcephaly can be masked by hydrocephaly, until a ventriculo-peritoneal shunt is implanted [4,23, CKH u.o.] or, exceptionally, by the presence of extremely large subarachnoid cysts [50].

**Figure 1:**
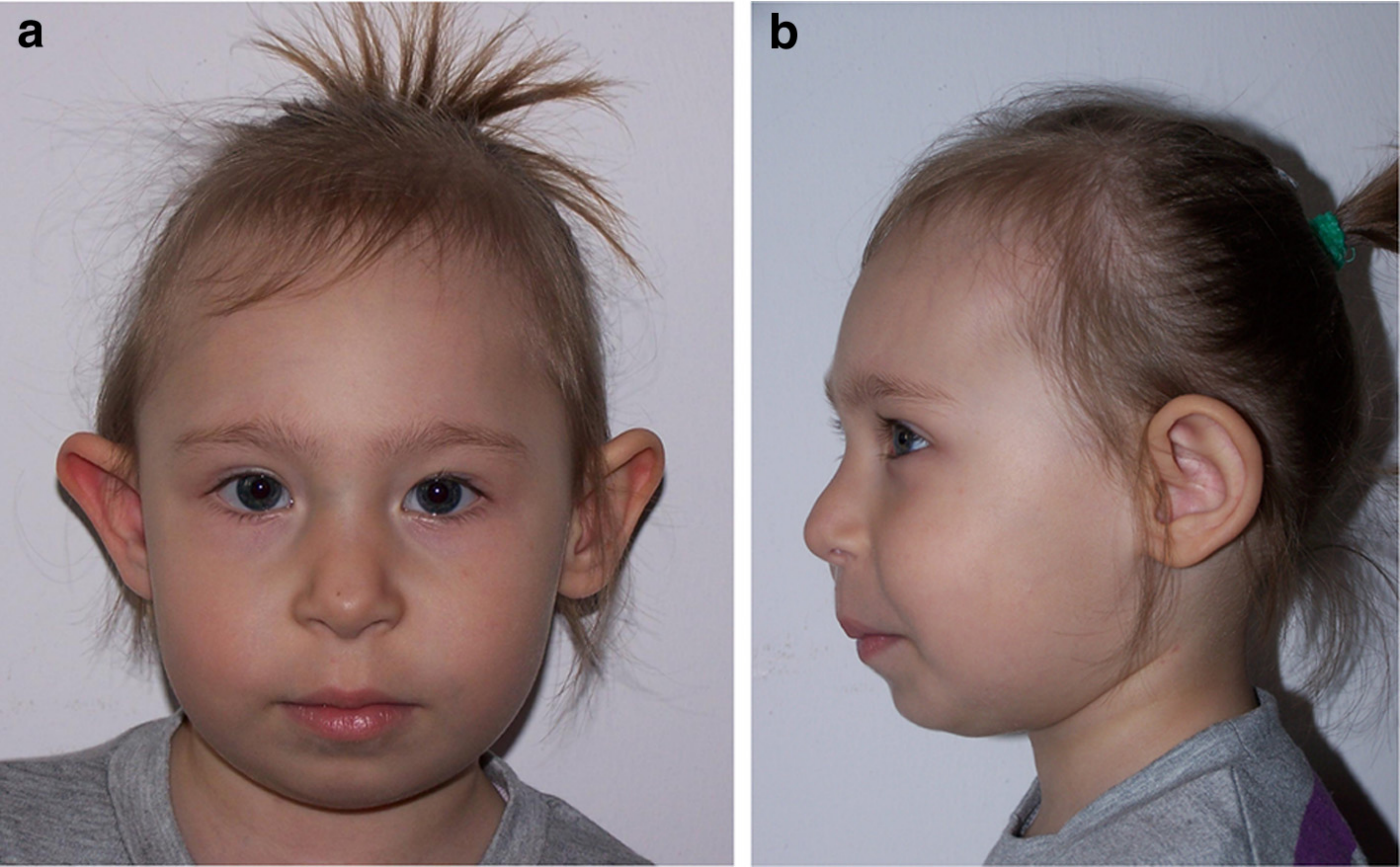
**(a, b). Facial phenotype in NBS: face (a) and profile (b) of a girl aged 3.5 years**. Note microcephaly, sloping forehead, small nose, receding chin, and relatively large ears.

The dysmorphic facial features are very similar among all patients with NBS and become more obvious with age. The prominent midface is emphasized by the sloping forehead and the receding mandible, which seems to be secondary to the underdevelopment of the cranium (Figure 1a, b). Other facial characteristics are more subtle and diverse, i.e. palpebral fissures are upwardly slanted in most patients, and the shape of the nose may be both long and beaked as well as upturned with anteverted nostrils. In individual patients, cleft lip/palate or choanal atresia have also been described [3].

## Neurocognitive and intellectual development

Despite severe microcephaly, developmental milestones are usually reached at normal times by the majority of NBS children [3,6,8,12,41,47,48]. It is striking to observe an infant or a toddler with severe microcephaly but with no motor problems and with very good comprehension. There are a few exceptions, such as a boy with schizencephaly [44] and monozygotic twin brothers with profound congenital microcephaly, poor gyrification of the brain and severe epilepsy [28]. Psychomotor development can be disturbed in individuals suffering from very early onset and severe infections [2,23,51].

Delayed speech development is common, and speech therapy is needed to correct articulation problems [CKH u.o.]. Intelligence was shown to vary from normal to mild or moderate mental retardation, but those reports were based on single psychological evaluations [2,3,12,40,41]. Long-term follow-up studies of a large group of 48 Polish patients indicate that cognitive development in NBS is a dynamic process: in early stages it generally stays close to the average (normal or borderline intelligence), but gradual deterioration is observed during school-age years [CKH u.o.].

## Growth pattern

The long-term study of over 70 Polish patients with NBS allowed observation of growth patterns from birth to adulthood, including puberty. Somatic development is delayed from the beginning [52]. The mean birth anthropometric parameters, including weight, length, head and chest circumferences are significantly lower than in a healthy population. Infants of both genders show a growth deficit until the age of 2 or 3 years when some gain of height and weight, but not OFC, is observed. In later stages of childhood and adolescence, differences in the growth pattern between the sexes become apparent: the growth spurt in boys is poor and is absent in girls. However, the adult mean height in over half of the girls and the boys with NBS is within lower normal ranges [52]. Thyroid and adrenal function, concentration of insulin growth factor-1 were assessed as normal and malnutrition syndromes were also excluded in the Polish studies referred to here [CKH u.o.].

## Sexual maturation

**Figure 2:**
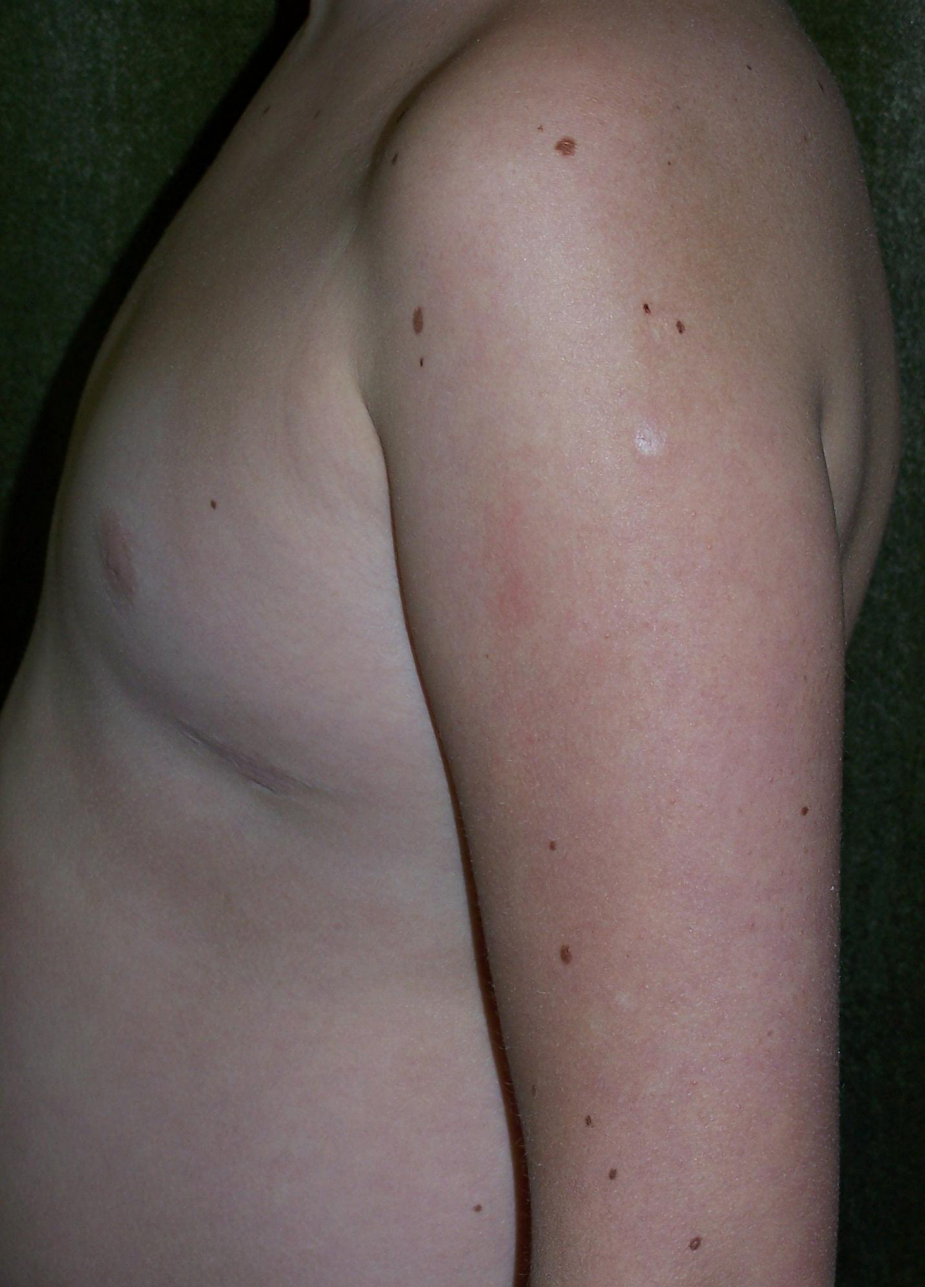
**Lack of breast development in a 16-year-old girl**. Note multiple pigmented nevi on the arm.

There is clear sexual dimorphism among patients with NBS in terms of pubertal development and concentrations of gonadotropins, [39,43,53,54]. In Polish studies, girls show no pubertal spurt and poor development of secondary sex characteristics (Figure 2) accompanied by low 17β-estradiol (E2) levels and high concentrations of follicle-stimulating hormone (FSH) and luteinizing hormone (LH) [53]. Moreover, gonadotropins showed a biphasic pattern, with mean values within normal ranges only in girls between 4 and 9 years of age, whereas in all remaining age groups, FSH values were significantly elevated, fulfilling the criteria of premature ovarian insufficiency. Additionally, ultrasound visualised small ovaries or solid streaks and a hypoplastic uterus [53]. In contrast, puberty in boys with NBS is initiated spontaneously and progresses similarly to healthy peers [54]. So far, there have not been any reports of NBS patients having offspring.

In contrast to the typical phenotype of NBS, it has been reported that nonsense mutations of the *NBN *gene can result in a far milder course of the disease manifesting only as a fertility disturbance [55,56].

## Congenital anomalies

### Brain malformations

Small brain size, particularly with underdevelopment of the frontal lobes, was found both intravital by MRI and at autopsy [15,57,58]. In each case, the brain was half or less than half the weight expected for age [3,59]. Communicating hydrocephalus has been diagnosed either at birth [48, CKH u.o.] or shortly after [4], as well as at autopsy in one patient and also after ultrasound on his foetus-sibling [23]. Aplasia or hypoplasia of the corpus callosum associated with colpocephaly and widening of the temporal horns of the lateral ventricles are the other most frequently reported anomalies of the central nervous system (CNS) [15,31,57,58]. Neuronal migration disorder (focal neuronal heterotopia) was documented in single cases in the form of schizencephaly [44], pachygyria [57] or partial lissencephaly [28]. On occasion, very large cerebral fluid collections in the form of arachnoid cysts were found on CT or MRI [40,50,57].

### Skeletal anomalies (minor)

Minor skeletal anomalies, such as clinodactyly of the 5^th ^fingers and partial syndactyly of the 2^nd ^and 3^rd ^toes are encountered in approximately half of the patients [12,47].

**Figure 3:**
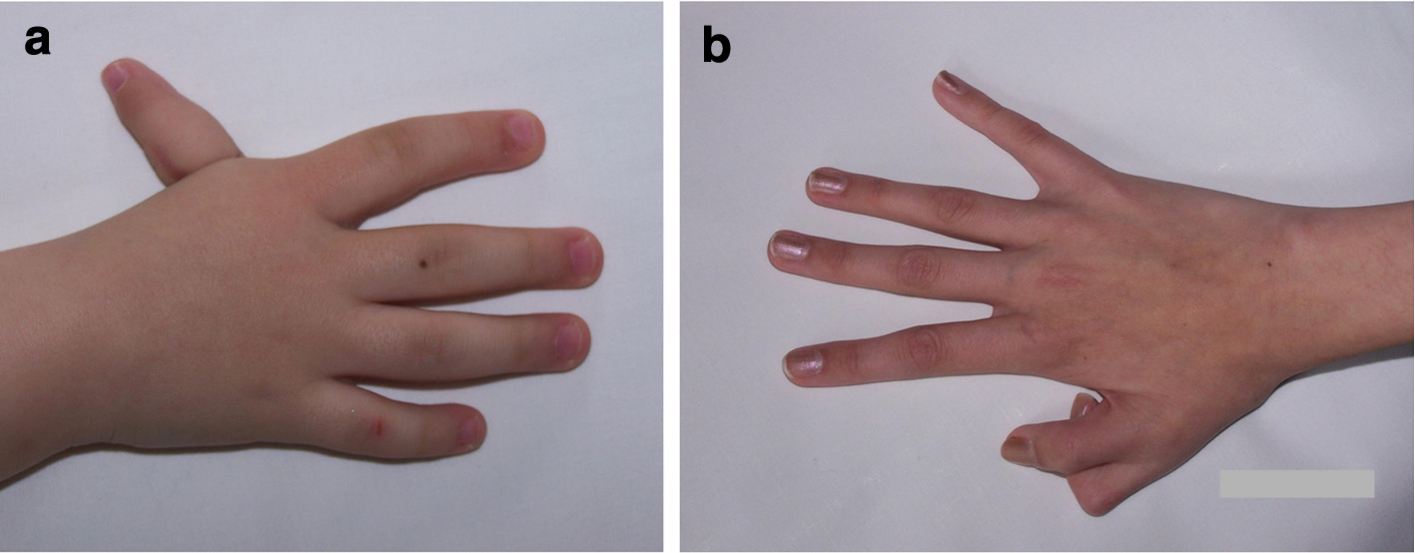
**(a, b). Hypoplastic and hypermobile thumb (a), and duplicated thumb (b)**.

Polydactyly, most frequently preaxial, occasionally also postaxial, as well as a hypoplastic or absent thumb were found in single cases [15,49], (Figure 3a, b). A radial defect, especially of the reduction type is relatively frequent in Fanconi anaemia (FA), another chromosomal instability disorder [60]. Hip dysplasia was found in a proportion of NBS patients [48], and single cases with agenesis of one rib [15] or the sacral bone were also described [48].

### (b) Skin and hair

*Café au lait *spots and/or vitiligo spots are the most frequently reported skin pigmentation anomalies observed in approximately 50%-70% of patients [12,34,47-49]. In three Polish patients, progressive depigmentation spreading all over the body was observed [49]. Multiple pigmented nevi (Figure 2) are present in a proportion of patients [39,45, CKH u.o.]; giant nevus was reported once [31].

In three patients, appearance of cutaneous, sarcoid-like granulomatous lesions localised mainly on limbs and face, which were refractory to treatment, preceded diagnosis of diffuse large B-cell lymphoma (DLBCL) by 1-6 years [32,35,49].

Hair in NBS is usually thin and sparse in infancy and early childhood but improves with age. Hair greying can appear as early as in the second or third decade of life [CKH u.o.].

### Genito-urinary system and anal area

Congenital anomalies of kidneys such as renal hypoplasia/aplasia, the horseshoe or double kidney, ectopic/dystopic kidneys are relatively frequent in NBS patients [6,12,23,31,34]. These defects can also be associated with urinary tract anomalies, such as duplication of the renal pelvis and ureter or hydronephrosis [2-4] and promote infections in the urinary system, particularly in patients with a defective immune defence.

Hypospadias, cryptorchidism, urethro-anal fistula were also found [44,46, CKH u.o.].

Anal atresia or hypoplasia was noted in at least six patients [8,31,61, CKH u.o.,].

## Predisposition to malignancies

Patients with NBS have a high risk for developing malignancy, the major cause of death in these individuals. Of all the chromosomal instability syndromes, the incidence of cancer in NBS patients is one of the highest.

By the age of 20 years, over 40% of NBS patients develop a malignant disease, predominantly of lymphoid origin. Non-Hodgkin lymphomas (NHL) of B and T cells are the most common, predominantly diffuse large B cell lymphoma (DLBCL), and T-cell lymphoblastic lymphoma (TLBL), however, Burkitt and Burkitt-like lymphomas are also encountered [62]. Data from the Polish Registry of NBS patients indicate that the distribution of lymphoma between B and T cell origins is approximately even (45% and 55% respectively) [63]. Hodgkin disease, prolymphocytic leukaemia, acute lymphoblastic leukemia (ALL) with precursor B cells or T cells, and acute myeloblastic leukemia (AML) have also all been described [3,18,20,35,64, CKH u.o.]. An extremely rare extranodal location of primary NHL has been described in a 6-year-old boy with NBS who developed pulmonary large B-cell NHL [17]. NHL arising in the paravertebral region was diagnosed in two young adults presenting with unexpected spastic paraplegia [CKH u.o.].

Brain tumours such as medulloblastoma [65-67], and rhabdomyosarcoma of extremely rare perianal localization have been reported to develop more frequently in NBS patients [24,31,44,61,63]. Single cases of papillary thyroid carcinoma, gonadoblastoma, glioma, meningioma, neuroblastoma, and Ewing sarcoma were also described [48].

An individual NBS patient treated for one cancer is at an increased risk for developing different consecutive malignant diseases of which lymphomas are the most common [62,63]. Morphology, immunophenotype profile and clonal Ig/TCR gene rearrangements performed in 2 cases confirmed the *de novo *development of the second lymphoma [64].

## Haematological disorders

Aplastic anaemia (AA), a disease characteristic for FA has been reported in at least three NBS patients of Slavic origin [18,68]. In one case, bone marrow aplasia was associated with EBV-related B-cell lymphoma [68].

Interestingly, Shimada et al. [69] reported a girl diagnosed with idiopathic severe AA at the age of 9, who was mosaic with a majority of cells homozygous for the *NBN *mutation I171V (c.511A < G; p.Ile171Val). The girl had neither microcephaly nor immunodeficiency characteristic of NBS.

Autoimmune complications such as haemolytic anaemia and a decrease in levels of the clotting factor V related to T-cell prolymphocytic leukaemia (T-PLL) were reported in a young adult with NBS [20].

## Predisposition to infections

The majority of patients suffer from infections of the respiratory tract, i.e. sinusitis, pneumonia and/or bronchopneumonia that result in bronchiectasis in some patients, and may even cause death due to respiratory failure [2,3,6,8,31,34,42,51]. As a consequence of severe frequent or persistent infections, in a few patients amyloidosis developed causing renal failure and death [18, CKH u.o.]. Other relatively common infections are: otitis media and mastoiditis, followed by urinary and gastrointestinal tract infections. Meningitis or encephalitis [23, CKH u.o.] and cavernous thrombophlebitis restricted to one hemisphere [16] were also observed in single cases.

In NBS patients, as in A-T, opportunistic infections are very rare [12,70], however, prolonged or repeated courses of antibiotic therapy may result in colonization by *Pseudomonas aeruginosa*. Oral *Candida *infections have been observed in approximately one third of patients in two large NBS groups followed for several years [31,71].

Tuberculosis was reported in three cases: a generalized type [18], a form restricted to the lungs and cervical lymph nodes [34], and as a primarily cutaneous form [36].

Viral infections, especially those caused by lymphotropic and/or hepatotropic viruses (EBV, CMV, HBV, HCV) may present a severe and chronic course, frequently accompanied by lymphadenopathy, hepatosplenomegaly and/or pancytopenia which may mimic lymphoma or leukaemia. Moreover, chronic viral stimulation of defective cells may lead to monoclonal malignancies such as B-cell and T-cell lymphomas [72,73]

On the another hand, there are also patients who, despite confirmed immunodeficiency, do not suffer from frequent infections and do not need immunoglobulin supplementation until adulthood or development of a malignancy [6,39,74, CKH u.o.].

## Immunodeficiency

A longitudinal follow-up study of 70 NBS patients has shown considerable variability in the immunodeficiency observed among different NBS patients, as well as in the same individual over the course of time. Systematic monitoring of immune biomarkers has shown that disturbances of the immune system are very heterogeneous and can be profound, with a tendency to progress over time.

### Cellular immunity

#### T cell abnormalities

Immunological studies reveal mild or strong lympho- and leucopaenia in nearly half of the patients with an evident decrease of immunocompetent cells [75], T cell immunity in NBS patients is characterized by a reduced absolute number of CD3+ T cells in the vast majority of patients, accompanied by a reduced number of CD4+ (helper) T cells (in about 90% of patients), with naive CD4+ T cells expressing the CD45RA isoform significantly decreased or practically absent. It is worth noting, that in some NBS patients with normal total T cell numbers, CD4+ T cell counts may be low [76]. The number of CD8+ T cells can be normal, elevated or markedly diminished, with a decreased ratio of CD4+/CD8+ (< 1.0) being observed in over 70% of cases. The number of natural killer (NK) cells (CD16+/CD56+) varies from normal to significantly increased with individual fluctuation evident over time [12,70,76,77]. In the great majority of patients, a shift towards the memory phenotype of CD4+ T-lymphocytes expressing CD45RO isoform is observed with a severe disruption of the CD4+ naive cell/CD4+ memory cell ratio.

#### B cell abnormalities

The absolute number of B cells (CD19+, CD20+) is reduced in 72%-75% of NBS patients and a normal count is observed only in about 12%-18% [6,12,70,73]. In some patients, however, the absolute number of B cells may be elevated even up to 1.4-2 times over the upper limit for age, despite significant deficiency of serum immunoglobulins and/or specific antibody response [70]. This may indicate an intrinsic defect of the B cell immunoglobulin class switching process (CSR) [78-81].

#### Functional defects of lymphocytes

The *in vitro *proliferative response of T lymphocytes to stimulation with phytohemagglutinin (PHA), anti-CD3 monoclonal antibodies and/or anti-TCR monoclonal antibodies, as well as B cells to stimulation with concanavalin A (Con A) is greatly reduced in most NBS patients [2,3,6,8,12,43,47,70,73,77,82,83]. In most studies, delayed hypersensitivity skin tests were negative [8,84].

### Humoral immunity

#### Total serum immunoglobulins

The humoral immunodeficiency in NBS patients is highly variable and ranges from agammaglobulinemia to a moderate reduction in the immune response. The most characteristic feature of the humoral abnormalities is deficiency of at least one or more immunoglobulin isotypes (Igs). Severe hypogammaglobulinemia with IgG < 2.0 g/l accompanied by a reduced concentration of IgM and significantly decreased or undetectable levels of IgA is found in 20%-24% of patients [12,43,70]. The most commonly reported defects are IgG4 and/or IgG2 deficiency, followed by deficiency of total IgG and IgA [6,12,70,73]. Although concentrations of total serum Igs (IgM, IgG, IgA) are found to be normal in about 20% of patients, it is worth noting that normal levels of total IgG can mask deficiency of IgG subclasses, mainly IgG2 and/or IgG4.

#### Specific antibodies

Similar to total Igs, both normal [41,44] as well as disturbed antibody responses to tetanus, *Haemophilus influenzae *type B, diphtheria, polio and hepatitis B vaccination have been reported [8,21,51]. Naturally acquired specific IgG antibodies to three pneumococcal polysaccharides (serotypes 3, 19, 23) were found in only 25% of NBS patients investigated, while in all other patients titres were undetectable or at a very low level. This finding correlated with markedly decreased levels of total IgG2. Similarly, most patients vaccinated against HBV did not develop anti-HBs antibodies of a protective IgG isotype or did not respond at all [26,70].

#### Monoclonal gammopathy and viral infections

One of the interesting findings in NBS patients were elevated and/or gradually increasing concentrations of total serum IgM [21,31,70,73,76], and these abnormalities were frequently accompanied by monoclonal proteins [70,82]. In a longitudinal study conducted on a cohort of Polish NBS patients a high frequency (about 38%) of persistent monoclonal gammopathy of both IgM (κ, λ) and/or IgG (κ, λ) isotypes was observed. However, it is important to notice that, in contrast to IgM gammopathy, decreased or even very low concentrations of total serum IgG can mask the presence of IgG gammopathy in these patients [73].

Genetic material of viruses with lymphotropic capacity was found in nearly 68% of Polish NBS patients, with a high prevalence of EBV infection (in 63% of patients) accompanied by monoclonal gammopathy in most of them. In some patients, HBV and/or HCV co-infection was present and in four cases, CMV infection was found [73].

#### Clonality of BCR/TCR gene rearrangements

Persistent clonal rearrangements of BCR and/or TCR genes in peripheral blood lymphocytes were identified in about 73% of NBS patients with, at the time, no clinical signs of lymphoid malignancy. This phenomenon showed evident progression over time in most patients and was frequently accompanied by increasing viral load, especially EBV, and preceded lymphoma diagnosis by as much as 3 years [73].

## Aetiopathogenesis

The most common hypomorphic mutation in *NBN*, the gene responsible for NBS, is a five base pair deletion in exon 6 which leads to two truncated fragments of nibrin: the expected 26 kD amino-terminal fragment (p26-nibrin), and, surprisingly, a 70 kD protein (p70-nibrin) which is produced by a unique alternative initiation of translation at a cryptic upstream start codon and represents the carboxy-terminal portion of the protein [85]. Null mutation of *Nbn *is embryonically lethal in the mouse [86,87] but expression of the carboxyterminal fragment, p70-nibrin, rescues both *Nbn *null mutant cells *in vitro *and, indeed, mice *in vivo *[88,89].

**Figure 4:**
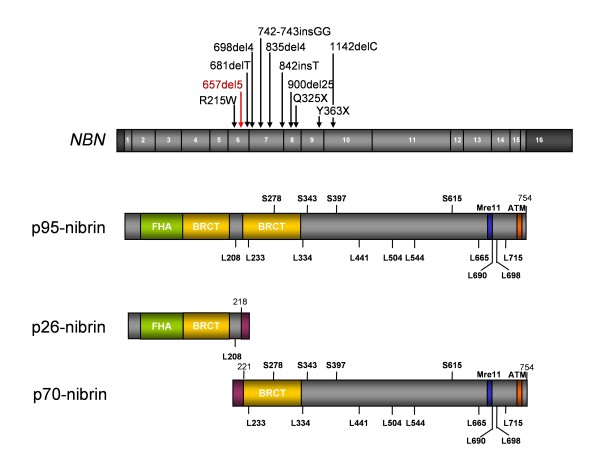
**The *NBN *gene and nibrin**. The exon structure of the gene is shown with the sites of mutations in NBS patients. The full length nibrin protein, with an apparent molecular weight of 95 kDa and the two protein fragments arising from the c.657_661del5 mutation are shown. The locations of serine residues targeted by the ATM kinase and acetylated lysine residues are indicated as are the relative locations of the FHA, BRCT, Mre11-binding and ATM-interaction domains.

Figure 4 shows the intron/exon structure of the *NBN *gene and its product, nibrin. Nibrin protein forms a trimeric complex with MRE11 and RAD50 (the MRN complex). In the amino-terminal portion of the protein, as defined by the c.657_661del5 mutation, there is an FHA domain and a BRCT domain, a second BRCT domain is located downstream from c.657_661del5 [11,90]. Such motifs are common in proteins which interact at phosphorylated residues and are frequently involved in cell cycle regulation [91,92]. At the carboxy-terminal end are domains required for association with the nuclease MRE11 and the kinase ATM [9,93]. *ATM *is the gene underlying A-T, a disorder with many features also found in NBS. ATM is the primary activator of the cellular response to DSBs and phosphorylates hundreds of target proteins in response to IR, including nibrin [94,95]. IR-induced phosphorylation of nibrin is further regulated by the deacetylase, SIRT1, which keeps nibrin in the hypoacetylated state necessary for efficient phosphorylation [96].

The trimeric MRN complex is rapidly translocated to the sites of DSBs after irradiation of cells and nibrin is essential for this process. The complex is a primary sensor of DNA DSBs and is required for the effective monomerisation and autophosphorylation of ATM after DNA DSB damage [97,98]. Clearly, this interaction between nibrin and ATM explains the considerable phenotypic and cellular similarities between A-T and NBS. The accumulation of MRN at damaged chromatin requires the MDC1 protein with which it interacts via the FHA domain of nibrin [99,100].

Nibrin is required not only for ATM activation but also for phosphorylation by the kinase ATR [101]. ATR (A-T and Rad 3-related) is activated by single-stranded DNA generated during excision repair or at stalled replication forks. The ATR signalling pathway is particularly sensitive to haploinsufficiency of genes implicated in its regulation, for example, heterozygous deletion of RPA1 and RFC2 lead to impaired ATR signalling in response to DNA damage [102].

The MRN complex is characteristically involved in both the repair of DNA damage and the activation of cell cycle checkpoints via ATM. Conditional *Nbn *null mutant fibroblasts are completely defective in the G2/M checkpoint following treatment with the radiomimetic drug bleomycin [88]. In the study by Difilippantonio and colleagues, murine B-cells with a conditional *Nbn *null mutation, and a transgenic human *NBN *gene, with or without the c.657_661del5 mutation, were examined. These *Nbn *null mutants had defective G2/M-phase and intra-S-phase checkpoints, comparable to those of ATM knockout cells [89]. ATM downstream phosphorylation targets were naturally also affected, with essentially no phosphorylation of Smc1, CHK2 or Brca1 in response to IR.

The cell cycle defects in null mutant cells were partially corrected by expression of the human *NBN *gene with the c.657_661del5 mutation and fully corrected by the wild type human gene. However, phosphorylation of nibrin was not necessary for restoration of cell cycle checkpoints [89]. In this connection it is noteworthy that the NBN mutation c.643C > T (p.Arg215Trp) leads to severe destabilisation of nibrin [28,103]. The level of full-length nibrin drops to less than 20% and results in strongly reduced activation of ATM [28].

Deficiencies in cell cycle checkpoints and disturbance of cell proliferation are possibly significant for the growth retardation of NBS patients. In addition apoptosis seems to be diminished when nibrin is absent or mutated. In two mouse models, disruption of the ATM interaction site in nibrin led to a severe apoptotic defect [104,105]. Since ATM is involved in IR-induced, p53-dependent and p53-independent, apoptotic pathways [106,107] the reduced apoptosis in the nibrin mutant cells reflects a failure in the activation of ATM. A study on human lymphoblastoid cell lines with different p53 status supports the view, that nibrin functions in IR-induced apoptosis via its role in activating ATM [108]. Whilst the consequences for cell proliferation are less clear, reduced clearance of heavily damaged cells is surely a contributing factor to the increased cancer risk in NBS [109].

Interestingly, p53 mediated apoptosis may play a role in microcephaly, a cardinal feature of NBS. Mutation of genes involved in neural proliferation is often associated with microcephaly [110] and indeed, generation of null mutation in Nbn specifically in developing neurons resulted in disrupted cerebellum development [111]. However, the relevance of this finding for NBS patients with a hypomorphic mutation of *NBN *remains to be established. A further contributing factor to microcephaly in NBS may be the ATR signalling mentioned above; microcephaly has been described as a general feature of defects in this pathway [102].

The deficiencies in immune response of NBS patients and the DSB processing defect in their cells suggest that immune gene rearrangements are inefficient in NBS. Although a gross V(D)J recombination defect was excluded [112,113], significant disturbances in the resolution of RAG-induced IGH breaks and compensatory proliferation of mature B cells have been recently reported suggesting a more subtle V(D)J recombination defect [114]. In addition, using conditional null mutant mouse models it has also been shown that nibrin is involved in immunoglobulin class switching [79,80]. After appropriate stimulation, less than 50% of null-mutant B-lymphocytes were able to switch from IgM to IgG1 or IgG3 [80]. In the humanized mouse model, where human p70-nibrin is expressed in the absence of murine nibrin, serum Igs showed a reduction of IgG1 and IgG3 isotypes reminiscent of NBS patients [89]. Furthermore, in B-cell and T-cell lymphomas isolated from these mice, complex translocations involving the IgH locus were found [89].

Mice heterozygous for an *Nbn *null mutation have a significantly higher incidence of tumours, importantly, in none of these tumours was the wild type allele lost or inactivated [87]. This suggests that *NBN *is a haploinsufficient tumour suppressor gene [115]. Indeed, it has now been proven that heterozygous relatives of NBS patients have a significantly increased cancer risk [116]. A gene dosage effect has been recorded for NBS patients too, with cancer occurrence lower in a subgroup of patients with above average p70-nibrin levels [117]. The individual variation in p70-nibrin levels is thought to reflect differences in its proteolytic turnover rather than differences in expression level [118].

In addition to characteristic translocations, lymphocyte chromosomes from A-T and NBS patients frequently show end-to-end fusions indicating telomere dysfunction [75,119]. Accelerated telomere shortening has been reported for both syndromes [120,121]. Data from mammalian cells suggest that one function of the MRN complex at the telomere may be C-strand resection [122]. Disruption of the MRN complex or inhibition of ATM led in normal telomerase-negative cells to telomere dysfunction [123]. Since telomere shortening triggers the entry of cells into senescence, the role of nibrin in telomere maintenance is likely to be highly relevant to growth retardation in NBS.

## Diagnosis, diagnostic criteria and methods

### Diagnosis and diagnostic criteria

Diagnosis of NBS is based on:

- characteristic clinical manifestations,

- chromosomal instability (spontaneous and induced),

- increased cellular sensitivity to ionizing radiation *in vitro*,

- combined immunodeficiency, cellular and humoral,

- mutations in both alleles of the *NBN *gene,

- complete absence of full length nibrin

#### Clinical criteria

Clinical diagnosis of NBS can be considered in children of both sexes presenting with the following characteristics:

a) Leading symptoms

- Microcephaly, usually present at birth,

- Recurrent respiratory tract infections

- Malignancies, especially of hematologic origin (NHL, ALL), but also solid tumours

b) Additional criteria

- Characteristic facial phenotype (sloping forehead, prominent midface, receding mandible); more evident with age,

- Mild growth retardation,

- Premature ovarian insufficiency

- General lack of neurological symptoms and good psychomotor development,

- Cognitive development better in early childhood (normal or border-line intelligence) with gradual decline.

Analysis of the family pedigree can also be supportive of NBS diagnosis if it indicates:

- sibling(s) with microcephaly or hydrocephaly,

- early deaths among siblings in the course of severe/recurring infections or malignancy,

- other family members with malignancies.

Early diagnosis of NBS is very important in order to avoid:

- severe recurrent infections, by employing appropriate prophylaxis,

- unnecessary exposure to radiation for diagnostic purposes,

- adverse reactions to radiotherapy for the treatment of malignant tumours.

### Diagnostic methods

**Figure 5:**
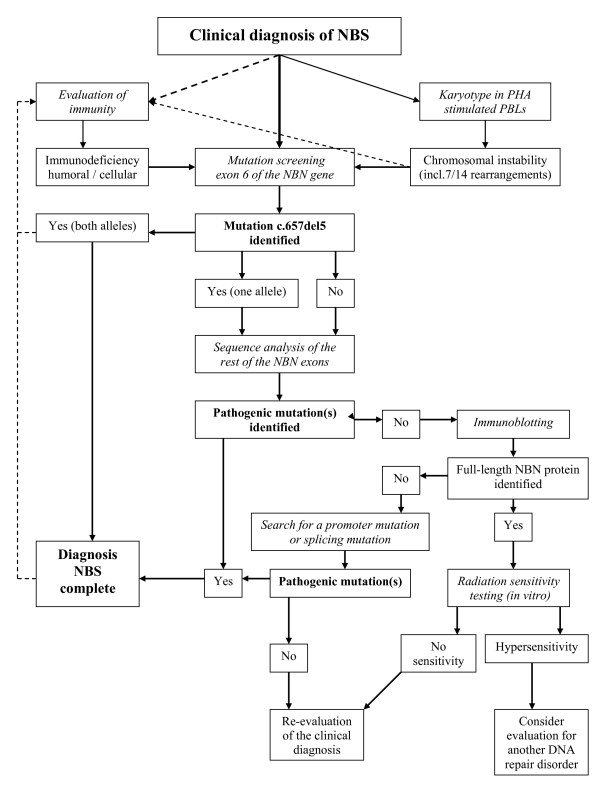
**Diagnostic flow-chart for NBS (see text for details)**.

The diagnosis of NBS is initially based on clinical manifestations and is confirmed by genetic analysis (Figure 5). Cytogenetics and molecular genetics can be utilized for the final diagnosis, the order of use may depend on the population origin of the patient. For Slavic populations, sequencing of exon 6 of the *NBN *gene is the method of choice, since over 90% of these patients carry the founder mutation c.657_661del5 on both alleles [11,12,18,25,34,37]. In other populations, or when molecular analysis is not possible, cytogenetic analysis could be the diagnostic method of first choice. For this, karyotype analysis is necessary, comparative genomic hybridisation is inappropriate [124,125].

### Cytogenetic analysis

#### Spontaneous chromosomal instability

Cytogenetic analysis of standard PHA-stimulated peripheral blood lymphocytes (T cells) allows detection of spontaneous chromosome instability, however, this may be hampered by the poor response of NBS patient lymphocytes to mitogens [3,6,8,44,50,65].

The constitutional karyotype is generally normal, however, a broad spectrum of abnormalities can be observed in 10-60% of cells. Among the aberrations most commonly found are open chromatid and chromosome breaks, aneuploidies, marker chromosomes, partial endoreduplication and structural rearrangements [2-4,6,8,14,15,34,39-45,51].

Inversions and translocations, involving two different loci in chromosomes 7 and 14 are particularly characteristic for NBS and are found in the vast majority of cases [28,51]. Significantly, the most frequently observed breakpoints are located in chromosome bands 7p13, 7q35, 14q11, and 14q32, which are the sites of the T-cell receptor genes (TCRG, TCRB, TCRA/D), and the human immunoglobulin heavy chain gene (IGH), respectively [126,127]. Such rearrangements are found in 10-35% of metaphases in NBS patient T lymphocytes [47] and in 5-10% metaphases in A-T [126,128], and also to a lesser extent in ATLD (1-8%) [129,130] and in NBSLD (1.5-4%) [131,132]. The most frequent aberration detected in NBS is inv(7)(p13;q35), followed by, in order of decreasing frequency, t(7;14)(p13;q11), t(7;14)(q35;q11), t(7;)(p13;q35) and t(14;14)(q11;q32) [6,42,47]. Other breakpoints in chromosomes 7 and 14 have been observed [133]. In addition, Stumm et al. found an increased frequency of other nonspecific translocations in both NBS and A-T using a three-color chromosome painting technique [133].

A few unusual cytogenetic cases have been reported. Der Kaloustian et al. described a 5-year-old boy with NBS and rhabdomyosarcoma in whom monosomies of almost all chromosomes and triradial figures were found in 64% and 16% of lymphocytes, respectively [44]. Recently Seemanova et al. [28] described severely affected NBS monozygotic twin brothers who did not present the hallmark feature of the disease, i.e. chromosomal instability. Neither chromosome breaks, nor translocations, were increased, and no rearrangements involving chromosomes 7 or 14 were found.

No increase of sister chromatid exchanges (SCE) was found in lymphocytes from any NBS patient so far investigated [2,8,39,43,44].

Chromosomal aberrations in lymphoblastoid cell lines (LCLs, EBV transformed B lymphocytes) and in fibroblasts are different from those observed in T-cells. Balanced and unbalanced translocations, isochromosomes, partial endoreduplications, aneuploidies, and supernumerary marker chromosomes are frequently found. Rearrangements of chromosomes 7/14, if present at all, are in the form of telomere fusions [42,43].

#### Induced chromosomal instability

The frequency of chromatid and chromosome breaks after treatment with IR or the radiomimetic drug bleomycin is increased in the majority of tested NBS cell lines as compared with normal reference cells, although not as much as in A-T [4,8,15,41-43,45,74]. Enhanced chromosomal breakage in lymphocytes/LCLs and fibroblasts clearly differentiates NBS cells from healthy cells [49] however, in rare individual patients, no increase of chromosome breakage was found [28,39].

Reaction to alkylating agents has been tested in cells from some patients, but results are equivocal with both insensitivity [43,44,48] and moderate hypersensitivity [39,50,51,134-136] reported. A positive DEB test contributed to misdiagnosis of NBS as FA in one family, and as a consequence bone marrow transplantation was performed. The patient was re-evaluated and a rare mutation in the *NBN *gene was found, resulting in the final diagnosis of NBS [76]. The molecular pathways of these two diseases clearly overlap [135,137].

### Radiation hypersensitivity and radioresistant DNA synthesis

Assays based on other consequences of the characteristic radiosensitivity of NBS cells, have been previously used to support diagnosis: the colony survival assay (CSA) [45,138] and the radio resistant DNA synthesis (RDS) assay [4,5,139]. The colony-forming ability of NBS LCLs after exposure to IR or radiomimetics *in vitro *is comparable to A-T cells and 2-4 times lower than in control cells [5,45,138,140].

High levels of RDS have been shown in the vast majority of NBS and A-T patients investigated [4-6,44,140] and reflects their impaired S-phase checkpoint [48]. After excluding mutations in the *NBN *gene, confirmation of hypersensitivity to IR using CSA or RDS assays strongly suggests evaluation for another DNA repair disease, such as ligase IV deficiency (LIG4 syndrome), severe combined immunodeficiency with microcephaly (NHEJ1 syndrome), NBS-like disease (NBSLD), or ataxia telangiectasia like disease (ATLD) [138].

### Molecular diagnosis

Molecular testing provides conclusive verification of the diagnosis. The majority of NBS patients identified to date are homozygous for the *NBN *mutation c.657_661del5 (p.K219fsX19): all but one patient in the Czech Republic, Poland and Ukraine; the majority in Russia; about 70% in the USA and many individual cases in other countries all over the world [11,14,18,21-23,27,30-32,34,35,141,142]. The remaining patients are either compound heterozygotes of the c.657del5 mutation and another unique mutation [11,18,28] or carry a unique mutation in both alleles [11,15,24,51,74,76]. All 11 known NBS-causing mutations are in exons 6-10 of the *NBN *gene.

Two further mutations were identified in exon 4 and 10 in French siblings with infertility [56] and one homozygous mutation in exon 5 in a Japanese child with AA [69]. None of these individuals presented the clinical features of NBS.

#### Testing strategy

Considering the frequency and spectrum of mutations, the most effective approach is to begin with screening for the founder mutation, c.657_661del5 (p.K219fsX19), in exon 6 of the *NBN *gene. In populations with a high frequency of this mutation [37] either Guthrie cards or EDTA blood can be used. If the founder mutation is not found or in only one allele, the next step is sequence analysis of the remaining exons. In the case of failure to identify a mutation or when the clinical diagnosis is ambiguous, it is worthwhile to establish a LCL and check whether nibrin is expressed by Western blot analysis. This testing strategy is shown schematically in Figure 5.

#### Western blot analysis

After generating a LCL, Western blot analysis allows detection of the protein and assessment for correct size. Finding full-length nibrin, after sequencing has shown only a wild type sequence, excludes the diagnosis of NBS. Testing sensitivity to IR *in vitro *is then advisable. If the cell line is hypersensitive, evaluation for another DNA repair disorder should be considered.

## Differential diagnosis

With some exceptions, NBS is usually diagnosed in childhood but rarely immediately after birth. Microcephaly, a hallmark feature of NBS, is a relatively frequent sign, which is also present in several hundred other inherited conditions as well as in many non-genetic diseases, such as CMV infections.

A combination of microcephaly, growth retardation and chromosomal instability links NBS with such diseases as NBSLD, LIG4 syndrome, NHEJ1 syndrome, FA, Seckel syndrome 1 and Bloom syndrome. Microcephaly and immunodeficiency are common features in NBS, LIG4 and NHEJ1 syndrome. Chromosomal anomalies which are characteristic for NBS and A-T patients, such as rearrangements between chromosomes 7 and 14 in T cells, can also be elevated in NBSLD and ATLD.

On the other hand, considering only the combination of chromosomal instability and immune deficiency, the closest relationship is found between NBS and A-T. These two diseases are caused by mutations in genes coding for proteins cooperating in the same DNA repair pathway, but presenting with distinct neurological manifestations [7].

The conditions most commonly confused with NBS are FA [51] and LIG4 syndrome [143].

All diseases considered in the differential diagnosis are inherited in an autosomal recessive manner and their phenotypic overlap with NBS is presented in Table 1.

**Table 1:** Disorders with chromosomal instability to be considered in the differential diagnosis of NBS

Condition	MIM	Gene	Clinical features	*In vitro *sensitivity to damaging agents
**NBS**	251260	*NBN* *(NBS1)*	Microcephaly, dysmorhic face, growth retardation (OFC more retarded than height); reduced fertility (hypergonadotropic hypogonadism in females); combined cellular and humoral immunodeficiency	IR, Bleomycin; Alkylating agents (MMC, DEB)--mild

NBSLD	604040	*RAD50*	Microcephaly, dysmorphic face, growth retardation (OFC more retarded than height); mild spasticity, non-progressive ataxia; normal puberty; no immunodeficiency	IR, Bleomycin

A-T	208900	*ATM*	Progressive cerebellar ataxia, oculomotor apraxia, bulbar and skin telangiectasia; (microcephaly only in exceptional cases); combined cellular and humoral immunodeficiency	IR, Bleomycin; alkylating agents (MMC, DEB) -mild

ATLD	604391	*MRE11*	Microcephaly (in some); cerebellar ataxia (late onset/slow progressive); oculomotor apraxia; no telangiectasia; no immunodeficiency	IR

DNA LIG4 syndrome	606593	*DNA LIG4*	Microcephaly, dysmorhic face, growth retardation (OFC more retarded than height); combined cellular and humoral immunodeficiency	IR, Bleomycin

NHEJ1	611291	*NHEJ1*	Microcephaly, dysmorhic facies, severe growth retardation; severe combined cellular and humoral immunodeficiency	IR (variable)

FA	227650	FANC*	Microcephaly (in some), growth retardation, skeletal abnormalities (radial defect); reduced fertility (hypergonadotropic hypogonadism in males); pancytopenia (progressive); no immunodeficiency	Alkylating agents (MMC, DEB); IR (mild)

Seckel syndrome 1 (ATR-SS)	210600	*ATR*	Severe microcephaly, severe prenatal and postnatal growth retardation; developmental delay, mental retardation; pancytopenia; no immunodeficiency	Alkylating agents (MMC); UV (moderate)

BS	210900	*BLM (RECQL3)*	Microcephaly, severe growth retardation (OFC retarded proportionally to height); no specific immunodeficiency	UV

WBS	613398	*DDX11*	Microcephaly, prenatal and postnatal growth retardation, deafness; developmental delay; no immunodeficiency	Alkylating agents (MMC)

*A-T *ataxia telangiectasia, *ATLD *ataxia telangiectasia like disease; *ATR *ataxia-telangiectasia and Rad3-related gene; *BS *Bloom syndrome; *DEB *diepoxybutane; *DNA LIG4 *DNA Ligase IV; *FA *Fanconi anemia; *IR *ionizing radiation; *MMC *Mitomycin C; *NBSLD *NBS-like disease; *NHEJ1 syndrome *severe combined immunodeficiency; *OFC *occipitofrontal circumference; *WBS *Warsaw breakage syndrome

*14 complementation group genes: *FANC-A, -B, -C, -D1/BRCA2, -D2, -E, -F, -G, -I, -J, -L, -M, -N, -O *(*RAD51C*)

## Genetic counselling

Parents of an affected child are obligate carriers of *NBN *mutations. In each pregnancy there is a 25% risk that a foetus will be affected. Siblings of a patient's parents are at 50% risk of being carriers. One exception to this rule is a NBS patient homozygous for the mutation c.657_661del5 in the *NBN *gene due to maternal isodisomy of chromosome 8 [30].

Heterozygous carriers of a *NBN *mutation do not present with any symptoms. However, in some population studies, a strong association with increased cancer risk was found for carriers of the founder mutation, c.657_661del5 [116,144-146]. This is of particular interest for Slavic populations, where the population frequency of the founder mutation is estimated at approximately 1/177 [37]. Parents, being obligate carriers, should be offered monitoring for cancer.

Identifying the mutations in both alleles in an index patient allows testing other family members at risk for the carrier state. There is not enough evidence from population studies to recommend such testing in childhood (i.e. among proband's sibs at risk).

## Antenatal diagnosis

Families at a 25% risk of having an affected child may be offered prenatal diagnosis if both disease-causing mutations in the *NBN *gene are known. Molecular analysis is the method of choice. Foetal DNA for molecular analysis can be obtained either by chorionic villus sampling (CVS) or by amniocentesis.

## Management including treatment

No specific therapy is available for NBS. Due to the specific basic defect underlying immunodeficiency and sensitivity to IR, patients with NBS require multidisciplinary medical management and long term follow-up. Specialised care provided by clinicians aware of these problems and of the natural history of the disease can prevent some complications, avoid unnecessary and excessive exposition to IR and take into consideration the high risk for malignancy. MRI and ultrasound examination are recommended as imaging techniques rather than CT scan or X-ray.

It is important that patients with NBS are under the care of one primary physician, preferably a paediatrician or a general practitioner, who is acquainted with the condition. Systematic (prophylactic) supervision by an immunologist and oncologist is recommended. Female patients should be under the care of an endocrinologist and a gynaecologist when they reach pubertal age. Psychological, social and educational support is essential for improvement of the quality of patients' life.

### Assessment, care and treatment of immunodeficiency

Monitoring of the immune system is extremely important throughout the whole life of a patient with NBS. Generally, it is recommended that the primary care physician should refer the patient to an immunologist. The immune biomarkers that should be evaluated in peripheral blood at the time of diagnosis and afterwards during follow-up are listed below.

The evaluation of immunological profile at the time of diagnosis:

- absolute number of T-cells (CD3+, CD4+, CD8+), T-cell subpopulations (CD45RA, CD45RO), B-cells (CD19+, CD20+), and NK-cells (CD16+ CD56+)

- function of T- and B-cells measured by proliferative response of lymphocytes to stimulation *in vitro *with mitogens and/or antigens (e.g. PHA, anti-CD3, anti-TCR, Covan A)

- concentration of total IgG, IgA, IgM and subclasses of IgG

- C3 and C4 complement components

- monoclonal proteins

- EBV DNA load, HBsAg (alternatively HBV DNA), HCV RNA (alternatively HCV RNA); the measurement of specific antibodies in NBS patients is unsuitable for diagnosis of virus infections

- BCR/TCR gene rearrangements in peripheral blood lymphocytes

The assessment of immunological biomarkers during control visits:

- absolute number of T-cells(CD3+, CD4+, CD8+) and B-cells (CD19+, CD20+)

- concentration of total serum Ig's

- monoclonal proteins

- EBV DNA load, HBsAg (if previously negative), HCV RNA (if previously negative)

- BCR/TCR gene rearrangements in peripheral blood lymphocytes

The assessment of immunological biomarkers should be performed once a year in patients who are in good clinical condition, and at 3-4 month intervals in patients who manifest evident progression of the immune system deterioration over time.

As in other combined immunodeficiency syndromes, NBS patients generally need gamma-globulin replacement therapy due to IgG deficiency. The overall consensus among clinical immunologists is that an IVIG or SCIG dose that maintains a serum IgG level over 5.0 g/l (at least 4-6 g/l) is desirable [147]. SCIG infusion therapy at home is an effective, convenient, and well tolerated alternative treatment modality, which gives significant improvement in the quality of life [148]. It is recommended that NBS patients should not be vaccinated with live bacterial or viral vaccines.

### Assessment, care and treatment of malignancy

Symptoms of lymphoid malignancies in patients with NBS and in the general population are usually similar but in immunodeficient patients can be mistaken for other conditions leading to fever, lymph node enlargement, loss of appetite and weight.

All diagnostic tests for lymphomas are the same as in the general population, however, the pathological and immunophenotypic picture may not always be characteristic and this requires evaluation by an experienced pathologist. In doubtful cases, classification may be facilitated by using molecular cytogenetic techniques [64,149,150]. This is crucial for the final diagnosis and assigning patients to appropriate general treatment protocols [151,152]; there are no specific treatment protocols designed for patients with lymphomas and NBS. It has been noticed that disorders such as A-T and NBS are associated with an increased risk for treatment-related toxicity [13,153]. Treatment-related deaths described in NBS patients are mainly from sepsis but death due to early anthracycline-induced cardiomyopathy was also reported [19].

Based on these observations it is common practice to modify treatment for NBS patients by limiting the doses of some chemotherapy agents (cyclophosphamide, ifosfamide) and even avoiding others (epipodophyllotoxins). The intensity of therapy is usually adapted to individual risk factors and tolerance [13,21,29,153].

It is most likely that modification of treatment by lowering the doses or omitting some anticancer drugs obscures final results. Administration of chemotherapy with doses greater than 80% of prescribed drugs was reported feasible and treatment related complications manageable [62]. Relapse of NHL remains a major cause of treatment failure [62,64].

There is new data emerging on successful treatment of NBS patients with refractory or relapsed lymphoma with hematopoietic stem cell transplantation (HSCT) [83]. Five out of 6 patients have restored T-cell immunity and are alive with a median follow up of 2.2 years. This suggests that HSCT should be considered for NBS patients since they present with consecutive episodes of lymphoma and correcting immunity may increase their survival rate.

Children with NBS can develop malignant tumours other than of lymphoid origin. Brain tumours in these patients have been described (medulloblastoma, glioma). Treatment of such patients should be restricted to chemotherapy only as it was reported that CNS irradiation resulted in radiotherapy-related death in 3 NBS patients [65-67].

## Follow up of growth and puberty

Endocrinological evaluation in NBS should include anthropometric monitoring of growth from birth and careful clinical evaluation of puberty in both sexes. Due to the marked radiosensitivity in patients with NBS, assessment of bone age based on hand and wrist roentgenograms should be avoided. Premature ovarian insufficiency is expected in most female patients with NBS, thus basal assays of plasma concentrations of FSH, LH, and E2 in females reaching pubertal age are recommended. Hormonal assays should be repeated in case of initially normal results, as the pituitary-gonadal axis may exhibit gradual dysfunction. Pelvic ultrasound is an auxiliary non-invasive examination, visualising the hypoplastic uterus and ovaries. Female patients with delayed or absent sexual maturation require the regular care of an endocrinologist, gynaecologist, or both. When hypergonadotropic hypogonadism is confirmed, replacement hormone therapy to induce secondary sexual characteristics, as well as to prevent osteoporosis, metabolic, cardiovascular and psychosocial sequelae must be considered when a patient reaches the appropriate age.

Despite normal puberty in NBS males, their sexual development and gonadal function should also be supervised periodically, particularly since cases of cryptorchidism and other anomalies of the genito-urinary system have been previously described [44,46, CKH u.o.].

## Psychologist/speech therapist

Delayed speech development is observed in many children, and speech therapy is needed to correct articulation problems.

Psychological assessment (IQ), before starting school education and then periodically every few years to give educational support is recommended because gradual deterioration of intellectual skills with age is observed in NBS individuals [CKH u.o.].

## Prognosis

The prognosis for patients with NBS and malignancies is unfortunately still poor. The largest series of 17 Polish NBS patients with NHL indicates that a cure is possible in individual patients and that the probability of survival is better for those with B cell NHL rather than T cell lymphoma. Thus, it is crucial to diagnose the disease as early as possible. Monitoring of monoclonal gammopathy, which in immunocompromised patients tends to evolve to lymphoproliferative diseases, might be a useful laboratory tool before serious complications of the disease develop. However, more data is required to confirm the predictive value of monoclonal gammopathy monitoring for early lymphoma diagnosis. Hematopoietic stem cell transplantation should be taken into consideration for patients with NBS and NHL, since correction of the immune system might help prevent further malignancies.

## Conclusions

Human diseases with deficiencies in DNA repair have been extremely helpful in elucidating the networks involved in the highly complex cellular response to DNA damage. This has often led to a deeper understanding of the aetiology of the diseases and had consequences for patient management, for example, in the case of NBS, the recognition that therapeutic and diagnostic radiation is to be avoided at all costs. It is to be hoped that further basic research will benefit patients even more. For example, examination of the cellular defect in patients with a particularly mild manifestation of the disease could potentially lead to the development of therapeutic strategies. Similarly, identification of modifying genes could help in prognosis and in optimizing conventional therapy.

## Competing interests

The authors declare that they have no competing interests.

## Authors' contributions

**KHC **conceived and designed the review, which was written together with **HG **(immunodeficiency), **BDB **(malignancies), **MAK **(growth and development, and endocrinology), and **MD **(aetiopathogenesis). All authors read, critically revised and approved the final manuscript.
